# “I'd like five of them”: the racialization and commodification of internationally recruited nurses in the German healthcare sector

**DOI:** 10.3389/fsoc.2025.1646906

**Published:** 2025-08-13

**Authors:** Tanja Gangarova, Johanna Kechout, Hans Vogt

**Affiliations:** German Center for Integration and Migration Research (DeZIM), Berlin, Germany

**Keywords:** nursing, racism, racialization, commodification, healthcare, Germany

## Abstract

While research in North American and Australian contexts demonstrates how encountering racism in healthcare is burdensome, the impact of racism on healthcare interactions in Europe, and particularly in Germany, remains underexplored. This paper draws on a study that examines the intersections of interpersonal, institutional, and structural racism in the professional experiences of internationally recruited nurses and integration managers in Germany who self-identify as “affected by racism.” The aim is to deepen our understanding of the forms, dynamics, and effects of racism within a healthcare sector increasingly shaped by economization. Employing an exploratory qualitative research design, the study is based on 21 semi-structured interviews and 10 participant diaries. Data analysis followed an iterative process, integrating inductive and deductive approaches within a framework of qualitative content analysis and emphasizing study participants' perspectives. The analytical framework developed in this paper addresses the intertwined processes of racialization and commodification and contributes to an empirically grounded conceptual understanding of recruited nurses' experiences of racism within nursing settings. This methodological approach reveals the subtle mechanisms through which racial inequalities are (re)produced and the ways that recruited labor is commodified in the German healthcare context. Findings suggest that these dynamics not only harm internationally recruited nurses, but also contribute to the invisibilization of racism within the sector. Furthermore, the analysis provides a nuanced account of racialized labor relations in transnational healthcare work and the global dynamics of care drain. In doing so, the paper identifies critical areas for institutional change and transformation, and it underscores the broader power structures that must be addressed in order to advance equity and justice in nursing.

## 1 Introduction

Reflecting on racism within the German nursing sector, a study participant—an integration manager who self-identifies as “affected by racism”—writes in her diary:

*The head of a ward informs me that she's heard that we'll soon be recruiting in Vietnam. She says: “I'd like five of them.”*^1^ *I then ask her: “You would like five nurses from Vietnam?” She answers: “Yes, the main thing is that they're Asian women.” No words*. (Manag 1, Diary, Pos. 13)^*^

This depiction sums up the various dimensions reflected in study participants' interviews and diary entries by capturing the intersecting processes of racialization and commodification of labor power that are faced by internationally recruited nurses from the Global South.^2^ Racialization—the marking of bodies as superior or inferior based on actual or ascribed biological, cultural, or ethnic characteristics (Essed, 2020; Grosfoguel et al., 2015)—and commodification—the capitalist reduction of labor power or even human beings to “objects of exchange” (Ashiagbor, 2021)—together lay the groundwork for extraction and exploitation in service of labor market demands in (and for the benefit of) the Global North. This further reflects a global order that is shaped by white supremacy and sustained through neo-colonial and racialized power relations (Grosfoguel et al., 2015). Additionally, it bears witness to Cedric Robinson's (1983) observation regarding the transnational nature of “racial capitalism”: “[t]here has never been a moment in modern European history (if before) that migratory and/or immigrant labor was not a significant aspect of European economies” (p. 23).

A striking and timely example of the deep dependency of Global North economies and societies on a migrant labor force is the nursing sector. The shortage of nursing staff in Germany—as well as in other Western countries—has led national states, hospitals, and private agencies to recruit nurses from the Global South (Hagey et al., 2001; McElmurry et al., 2006; Batnitzky and McDowell, 2011; Kaelin, 2011; Germack et al., 2015; Hanrieder and Janauschek, 2025). Various programs to recruit foreign nurses have been established in the German context, including the Western Balkans Regulation (in place since 2016) and the Triple Win program, which has enabled the recruitment of nurses from outside the EU (for example, Tunisia, India, the Philippines) since 2013 (Agentur für Arbeit, 2025; Mediendienst Integration, 2025). In addition to the ethical concerns associated with the extraction of healthcare professionals from post-colonial societies (McElmurry et al., 2006; Bradby, 2014) and the significant loss of training investments and skilled labor for their countries of origin (Kaelin, 2011), it is important to note that the recruited nurses are often placed in precarious working positions, which frequently involve roles that are considered undesirable by nurses from the host countries (Iheduru-Anderson et al., 2021). Ahlberg et al. (2019) observe this phenomenon when they refer to the notion of “care drain” (Kaelin, 2011), which they define as “a double bind for those migrating to escape the structural violence of poverty, joblessness, and inequalities in source countries, only to experience continued structural violence through institutional racial discrimination and mistreatment in destination countries” (p. 5).

While an established body of literature, largely from the North American and Australian contexts, demonstrates that racialized healthcare professionals (including nurses) face various forms of racial discrimination at their workplaces (Hamed et al., 2022), the impact of racism on healthcare professionals remains gravely understudied in Europe, particularly in Germany. A limited number of studies from recent years (Ritter, 2023; Richter, 2022; Lugert-Jose, 2022, 2023) provide selective insights indicating that racism in the German nursing sector can negatively affect not only the health and professional development of nursing staff, but also the quality of healthcare overall, and that nurses are left alone to cope with harsh and unbearable working conditions (Bühler, 2024).

The study presented in this paper contributes to the research field by examining the intersections of interpersonal, institutional, and structural racism in the professional experiences of internationally recruited nurses and integration managers in Germany who self-identify as “affected by racism.” It seeks to deepen our understanding of racism's forms, dynamics, and effects in close relation to the economization of the healthcare sector. Our study employs an exploratory qualitative design, drawing primarily on 21 semi-structured interviews and 10 participant diaries. “Exploratory” in this context means that we sought to take initial steps toward investigating a field that has remained largely understudied from an explicitly racism-critical lens, particularly in Germany.

The analytical framework developed in this paper seeks to address processes of racialization and commodification, as well as their mutually reinforcing intersections, and contributes to an empirically grounded conceptual understanding of internationally recruited nurses' and integration managers' experiences of racism in nursing and beyond. The methodological approach therefore illumines the subtle mechanisms that lead to the (re)production of racial inequalities in the nursing sector and the commodification of recruited labor power in Germany. Thus, our study responds to scholarship that critiques current research on racism in healthcare for its lack of theoretical focus on processes of racialization (Hamed et al., 2022; Nazroo et al., 2020). Another added value of our study lies in having taken additional and more differentiated steps in documenting the commodification effects produced by racialization in the German healthcare context. This is an insight that emerged organically from our empirical data.

Our paper is delineated as follows: We begin with a brief introduction, followed by an overview of racism in the (German) nursing sector. Next, we describe our theoretical framework and methods applied in the study. This is followed by a presentation of our findings. In the final section, we locate the results theoretically and empirically in the wider body of literature and offer some concluding thoughts.

## 2 Racism in (German) nursing

Although scholarly attention to racism encountered by racialized healthcare professionals across diverse geographical contexts has only recently gained momentum, a scoping review by Hamed et al. (2022) indicates that nurses—who represent the most extensively studied group—face both overt and covert forms of racism from both patients and colleagues. For instance, a recent qualitative study conducted in Canada by Beagan et al. (2023), which examines the intersections of interpersonal, institutional, and structural racism in the experiences of nurses, highlights racism in the form of comments and actions by patients, but more significantly through lack of support from colleagues and managers. Similar findings have been reported in Finland (Wesołowska et al., 2020) and the UK (Stevens et al., 2012), where racialized nurses experience bullying and discrimination from fellow staff members. (Estacio and Saidy-Khan 2014) further document experiences of racial microaggressions among migrant nurses in England, alongside institutional barriers that hinder career progression and access to further training. Institutional and structural racism have been identified as significant factors, as they contribute to increased scrutiny of, disproportionate workloads for, and persistent negative assumptions—for example, regarding professional incompetence—about racialized nurses in Canada (Beagan et al., 2023) and in the United States (Likupe and Archibong, 2013). Experiences of racism can result in elevated stress levels, negative mental health outcomes (Hagey et al., 2001; Likupe and Archibong, 2013), pervasive feelings of anger and frustration (Estacio and Saidy-Khan, 2014), and emotional depletion (Cottingham et al., 2018). In various national contexts, these experiences are not discussed at the workplace, frequently due to fears of adverse consequences (Ahlberg et al., 2022; Beagan et al., 2023; Hamed and Bradby, 2023). Racialized nurses report being left to choose between remaining silent, resisting (often at personal cost), assimilating, or bolstering their credibility through extra work (Beagan et al., 2023; Ahlberg et al., 2022). According to the aforementioned review, the impact of racism on healthcare interactions remains significantly understudied in Europe, particularly in Germany. Furthermore, existing research on racism often lacks a robust theoretical focus on the processes of racialization, making it difficult to conceptualize racism and to understand how racial inequalities are (re)produced in healthcare encounters (Hamed et al., 2022).

This lack of data on racism and healthcare in Europe may be explained by the fact that the usage of racial categories in data collection in general was rejected in European countries (except for the United Kingdom) following the Second World War, on the grounds that using these in official data is inherently racist (Möschel, 2011). For instance, in the German context, public and institutional engagements have long externalized racism temporally, socially, and spatially. Often framed as a phenomenon of the National Socialist past, racism is frequently displaced onto right-wing extremism and thus seen as a marginal issue (Bojadžijev et al., 2017). Additionally, by portraying racism as primarily a problem of former colonial powers or the United States, German discourse has obscured its own colonial history and its ongoing impact on contemporary racialized structures and practices (Bojadžijev et al., 2017; Salem and Thompson, 2016).

Due to the aforementioned denial of racism in Germany (see also Lentin, 2008), research on professional healthcare work in the context of migration and/or employment has, for a long time, predominately framed problems through the lens of “cultural differences” (cf. Ritter, 2024a, p. 16). An explanatory approach of this sort risks promoting racist categorizations and remaining silent about existing structures, which should instead be problematized as risk factors in any context (Velho, 2010). Research that adopts an explicitly racism-critical perspective on the topic has, however, remained marginal to date (cf. Pütz et al., 2019; Kontos et al., 2019; Rand and Larsen, 2019). Nonetheless, recent studies have begun to address racist attributions and devaluations in everyday professional healthcare work, in relation to both healthcare workers and patients (Domenig, 2021; Richter, 2022; Lugert-Jose, 2022, 2023). These studies conclude that experiences of racist exclusion have diverse and profound psychological and work-related consequences, and that these experiences may indirectly impact the quality of care and patient safety (cf. Richter, 2022). There are only a few studies that explicitly address racism in eldercare (cf. Ritter, 2024a,b; Ulusoy and Schablon, 2020). For example, (Ritter 2024b) examines the interconnections between racism and economization in eldercare by analyzing how racist exclusionary practices against migrant care workers are reinforced by economic pressures. While these studies provide an important starting point, the widespread and severe racist exclusions experienced by nursing professionals underscore the urgent need for further research from an explicitly racism-critical perspective—one that also considers the growing influence of neoliberal logics, which can intensify such exclusions in nursing contexts.

This paper addresses this gap by focusing on the intersections of interpersonal, institutional, and structural racism, as well as the interrelation between processes of racialization and commodification in the professional experiences of internationally recruited nurses. Since racism is considered a silent and silenced phenomenon in European healthcare (Bradby et al., 2019), we hope to contribute to breaking that silence by advancing the conceptual understanding of racism in nursing and by drawing attention to areas in need of change and transformation.

## 3 Methods and material

### 3.1 Theoretical framework

In this paper we employ a theoretical triangulation approach, integrating multiple perspectives—specifically theories of racism and neo-colonial theory—in order to more comprehensively capture the complexity of the phenomena under study. We draw on Philomena Essed (2020) conceptualization of racism as the systematic “creation of hierarchies of worthiness attached to groups of people identified as different in terms of (attributed) racial or cultural (ethnic) factors. It is a historically anchored ideology, structure, and process, whereby one racial or ethnic group privileges its members on the basis of ascribed, preferred values and characteristics in order to legitimize the disadvantaging of other groups. These values and characteristics are used to assess the worthiness of human beings and ways of being in terms of related degrees of entitlement to ‘be,' to be validated, and to develop” (Essed, 2020, p. 448). These global hierarchies have been politically constructed and historically reproduced over centuries to serve the interests of an imperialist-colonial, capitalist, patriarchal, and Western-centric world order (Grosfoguel et al., 2015). As such, they are deeply embedded within political, legal, and institutional structures. Another foundational theoretical lens for analyzing these dynamics is provided by Frantz Fanon in *Black Skin, White Masks* (2008), where he introduces the concept of the “zone of non-being.” Individuals and groups perceived as existing outside the “zone of non-being” are socially recognized as full human subjects and granted access to the complete spectrum of human and citizenship rights (including labor rights). In contrast, those situated within the “zone of non-being” are dehumanized—their humanity is either denied or fundamentally questioned. Racism, in this sense, constitutes a hierarchy of worthiness, organized along the boundary of what is considered fully human (Fanon, 2008). In a complementary theoretical framework, Robinson (1983) emphasizes that this hierarchy of worthiness is not incidental but lies at the core of “racial capitalism,” a concept that underscores how processes of capital accumulation have always been entangled with racial differentiation and domination. Such an approach (the combination of theories of racism and theories of neo-coloniality) to racism enables the exploration of neo-colonial relations, rather than post-colonial ones, and follows a social vision of decoloniality—confronting the persistent colonial logics and structures as they exist in the present day. It is in view of this background that we use the term commodification to describe the operation of a capitalist logic that objectifies human beings and labor power—particularly care work—through racialized (and gendered) global labor hierarchies (Fraser, 2016; Ashiagbor, 2021).

Additionally, we employ the framework of interpersonal, institutional, and structural racism delineated by Nazroo et al. (2020). Their framework is particularly helpful, as it pays attention to the micro, meso, and macro dimensions of racism and highlights the dynamic interconnections between these levels of analysis. Interpersonal racism refers to individual interactions and the routine reproduction of racist knowledge in everyday workplace encounters (Essed, 1991), including subtle, often normalized verbal and non-verbal assaults known as racial “microaggressions” (Sue, 2010), which help sustain institutionalized Whiteness. Institutional racism manifests through organizational practices, policies, and procedures that, while appearing race-neutral, systematically advantage white people and disadvantage racialized people. Structural racism encompasses the broader social arrangements that produce unequal access to financial, material, political, and symbolic resources. It also includes ideological dimensions that justify and maintain these inequities. Dominant beliefs about expertise and authority are shaped by structural racism, which legitimizes white ways of knowing and being and systematically marginalizes other ways of knowing and being (Dotson, 2011; Mills, 2007).

### 3.2 Recruitment and data collection

At the beginning of the study, the authors conducted a manual analysis of the existing national and international literature on racism, labor, healthcare, and nursing. This was followed by preliminary interviews with experts in the field of nursing and with nurses of color. These exchanges were documented in the form of handwritten minutes, and they served to gain initial access to the field, to identify problem areas, to refine the analytical understanding of the different levels of racism, and to delineate the research questions with greater precision.

The recruitment of twenty-one study participants (eighteen nurses and three integration managers) was supported by existing professional and community networks of the research team and advisory board members. These networks included nursing schools, hospitals, civil society organizations, nursing students, and engaged nurses of color. The participants were recruited via flyers and an open inquiry letter (in both German and English) addressed to nurses, nursing assistants, and integration managers who self-identify as “affected by racism.”

A combination of selective and convenience sampling was used to approach study participants; the aim was to capture a broad spectrum within the community under study. Considered characteristics of the nurses included gender (nineteen women, two men), age (ranging from 22 to 55 years), country of origin (Germany, Brazil, Mexico, Cuba, the Philippines, Cameroon, Morocco), German citizenship status, and type of residence permit. Different places of work and residence, across several German federal states, were also considered. Nearly all of the nurses have been living and working in Germany for 6 months to 4 years, and typically without the accompaniment of family members.

Although some study participants were still in the process of obtaining formal recognition of their professional qualifications in Germany, we generally use the broader term “nurse” in this article. The official professional designation for a fully trained and recognized nurse, however, is *Pflegefachperson* (nurse). Prior to receiving official recognition and certification as a *Pflegefachperson*, internationally trained professionals are typically employed and remunerated under the designation of *Pflegefachassistenz*.

The integration managers are women and hold German citizenship. Two of them work independently across Germany as freelancing consultants for hospitals and umbrella organizations. The third is employed as both a registered nurse and an integration manager at a large German hospital. The perspectives of the participating integration managers offered in-depth insights into structural and institutional dynamics for two main reasons: their extensive professional experience, combined with a comparative and reflective outsider's perspective on various recruitment contexts across Germany, enriched the analyses; their relatively secure social and legal positions— stemming from factors such as professional status, institutional embeddedness, and German citizenship—enabled them to articulate their observations and experiences more freely and without existential risk.

The German healthcare sector encompasses a wide range of domains, including political frameworks and legislation, the insurance system, public health services, NGOs, and counseling centers, which can be conceptually structured within a triangular relationship: service recipients (the general population or patients), service providers (counseling centers, hospitals and medical professionals), and service funders (state and health insurance companies). This study focuses specifically on nursing professionals working in hospitals—that is, in the field of acute inpatient care.

The authors designed an interview guide to facilitate but not determine discussions (Kallio et al., 2016). The semi-structured interviews started with contextual questions before moving on to questions related to racism (Seidman, 2005). Study participants were initially asked about their educational background and their motivation to become a nurse or an integration manager. The interview ended with debriefing in order to reflect on the interview and to provide contacts to counseling centers if needed. The interviews were conducted as video conferences in two languages (German and English), between July and September 2024, and lasted between 30 and 70 min. Each of the authors conducted seven interviews. The recorded interviews were transcribed, anonymized, stored, and coded by using the MAXQDA analysis software (VERBI Software, 2024). Integration managers interviewed in the study are referred to as Manag 1 to 3, while internationally recruited nurses are quoted as Int 1 to 18.

Following the interviews, all participants were invited to take part in a follow-up diary study. The diary method enables participants to reflect on their nursing shifts in an open-ended and relatively unfiltered manner. Data generated through diaries offer valuable insights into processes of reflexivity (Rosenberg, 1990) and capture more real-time experiences (Theodosius, 2008) that are often difficult to access through interviews. The ten nurses and integration managers interested in the diary study were asked to reflect on both positive and negative experiences encountered during and after their shifts. They were further encouraged to describe the individuals and factors that influenced their emotions and thoughts, as well as how they responded to these experiences. Participants were not asked to specifically reflect on experiences related to race/racism—however, racism as a research topic was indirectly predetermined by the interviews. The diary entries were anonymized, securely stored, and subjected to the same coding procedures as the interview transcripts. To mitigate epistemic exploitation—that is, the uncompensated and emotionally taxing epistemic labor often extracted from marginalized individuals (Berenstain, 2016)—participants were compensated 30 euros for the interviews and 100 euros for completing the diaries. This approach acknowledges, even if only symbolically, not only the value of their time and emotional labor but also their intellectual expertise.

During data collection, one of the interviewees provided anonymized documents that served to complement the interview by illustrating it through an institutional lens and literally documenting it. The documents included several employment contracts and rental agreements issued to the recruited nursing personnel, supplementary agreements to employment contracts, and correspondence from relevant authorities and employers, such as hospitals.

### 3.3 Advisory board

To ensure the methodological quality of the assessments and evaluations, an advisory board was convened. It consisted of four independent members who are experts in the field. Two of the members previously worked as nurses and self-identify as “affected by racism.” Throughout the entire research process, the research team was supported by the advisory board members, who provided feedback on the research questions, study design, preliminary findings, and research dissemination plan.

### 3.4 Data analysis

The data analysis developed iteratively, with inductive and deductive steps complementing one another. An initial coding scheme, based on the authors' reading of the interviews and diaries, was constructed. This coding scheme was applied to all interviews and diaries (each author applied it to seven interviews and all ten diaries), and the results were compared to inform modifications to the scheme. Core categories (including subcategories) were defined and redefined, with all three authors engaged in a continuous discussion throughout the analytical process. This collaborative approach, which aligns with the concept of “investigator triangulation” (Denzin, 2017), was employed to enhance the trustworthiness of the analysis and to mitigate potential single-coder bias. In addition, thematic memos were constructed during iterative readings of the transcripts to capture connections across different codes and the potential relevance of each transcript to the concepts of interest. Analysis moved from data to theory and back again, focusing on codes such as experiences of racism on different levels (interpersonal, institutional, structural/systemic), effects of racism, responses to racism, and agency. Subsequent to the initial thematic summaries and memos, both the interviews and the diaries were subjected to a more detailed content analysis, with a focus on the participants' perspectives (Mayring, 2019). The analysis was conducted in the original language of the interviews and diaries in order to stay close to the data. Quotes were translated into English only after the article was drafted. The quoted passages in this article that we (the authors) translated from German are indicated by asterisks (^*^). Feedback loops with the advisory board members were integrated into the process of analysis in order to further diversify the epistemic standpoints from which data was interpreted and to confront othering in data interpretation and data representation (von Unger, 2022). Overall, the study employed a triangulation of interview and diary data, in line with Flick (2011)), while the documents served primarily to illustrate and contextualize participants' statements, consistent with the concept of participant-driven elicitation (Guillemin and Drew, 2010). Triangulation addresses the complexities inherent in research by incorporating diverse viewpoints (without denying the value of individual accounts as such or assuming a form of completeness). By integrating potentially conflicting perspectives, it enhances the validity and robustness of the findings.

### 3.5 Ethical considerations, reflexivity

Ethical approval was obtained from the Ethical Review Committee of the DeZIM-Institute (approval no.: EK 07/2024). All participants received verbal and written information on the study and on the way in which their interview and diary statements would be treated. All participants gave informed consent in verbal and written form and received explanations of how we would safeguard their confidentiality and anonymity. They were also informed about the voluntary nature of their participation. Given the sensitivity of the research topic (experiences with racism), participants were provided with contact details for consulting centers where they could contact a therapist or psychologist if they wished. They were additionally given the contact details of the researchers, in case they had additional questions or inquiries. The collection and the analysis of the aforementioned anonymized documents were subsequently communicated to the ethics committee and received approval from the data protection officer of the research institute.

To recognize power relations both within the research team and in relation to participants, spaces for reflexivity were available throughout the research project. These were facilitated through feedback loops within the core team and with the advisory board. The research team consisted of one person identifying as a white cisgender woman, one as a queer, migrant cisgender woman—who is professionally educated as a diversity trainer and describes being perceived as Western Europe's “incomplete Self” (Todorova, 1997), or not white enough—and one as a white, cisgender man. This composition facilitated mutual learning within the research team. Furthermore, the reflexive process—particularly the feedback loops with advisory board members—enabled us, as researchers, to critically reflect on our own biases during data analysis, especially in relation to institutional Whiteness and our positionality within Western academia.

## 4 Results

In the following sections, we present a synopsis of the study's partial results, which we have divided into three sections: (1) Global Framing, (2) Institutional Racism, and (3) Interpersonal Racism. The primary objective of this categorization is to enhance narrative clarity without losing sight of the fact that the three levels are deeply interrelated and inseparable. It is imperative to acknowledge that each quotation inherently reflects elements of all three dimensions. Thus, the connection of global, institutional, and interpersonal racial injustices in mutually reinforcing intersections creates a structure that facilitates the (re)production of racist knowledge, racist practices, and racial inequalities/injustices. Moreover, it enables the emergence of a formidable power that often goes unnoticed, giving rise to, legitimizing, and perpetuating exclusion, oppression, and exploitation.

### 4.1 Global framing

*The head of a ward informs me that she's heard that we'll soon be recruiting in Vietnam. She says: “I'd like five of them.” I then ask her: “You would like five nurses from Vietnam?” She answers: “Yes, the main thing is that they're Asian women.” No words*. (Manag 1, Diary, Pos. 13)^*^

As noted in the introductory section, this quotation—taken from the diary of an integration manager in a hospital setting—illustrates how the head of a hospital department approaches the recruitment of nurses as a transactional process, treating them as quantifiable goods. The devaluation associated with the statement “*I'd like five of them”* is reinforced by the accompanying remark “*the main thing is that they're Asian women.”* This specification of a group serves as a generalization (homogenization), collapsing distinctions between Vietnamese and other “Asian women” into a single, undifferentiated category. In doing so, it implicitly ascribes certain attributes of employability to this purportedly/presumed homogeneous group. Concurrently, “Asian women” are regarded as fungible commodities that can be discussed in quantities. Our data suggest that this dehumanization, as an integral mechanism of the racialization process, is foundational to enabling the commodification of care workers recruited into the sector. Accounts of other study participants further illustrate how they are devalued in the context of racialized global relations and reduced to their labor power, which is commodified through capitalist logics.

The above quote is particularly significant, as it demonstrates how the perspective of the head of a German hospital ward is entangled with global dynamics and historically rooted narratives of racialized labor exploitation. This entanglement is reflected in the accounts of other interviewees. It articulates key dimensions of a global care drain, providing an important framework for the analysis of the empirical material.

*But nowadays, I think, in the Philippines, they are really in need of nurses because most of us, we already work abroad […] or some others have changed profession or career*. (Int 11, Interview, Pos. 10)

Similarly, the account of Int 11, another female study participant, illustrates how the phenomenon of care drain can be understood as the extraction of resources from countries of the Global South by richer, post-industrial countries of the Global North or the West. Conversely, care drain is associated with conditions in Germany.

*Yes, and so that's also a good example. The woman […] who, because she has not passed the exam, the clinic simply separates from her and puts her on the street. You can't treat people like that. That is a mother, she has three children, she is here in Germany alone, and then you simply put her on the street, and then the next ones are taken, right?* (Manag 3, Interview, Pos. 20)^*^

As suggested by this quote from Manag 3, a self-employed integration manager, the living and working conditions experienced by study participants in Germany are often marked by precariousness and exploitation. Within the framework of bilateral state agreements, individuals are brought to Germany by recruitment agencies under the promise of a “better life” (Hanrieder and Janauschek, 2025). However, they are subjected to a commodifying treatment that reduces them to objects, marginalizes their agency, and reinscribes them within global hierarchies of worthiness. In the context of recognition processes and exploitation logics, study participants' professional skills are often devalued through practices of “deskilling,” thereby subjecting them to intensified financial and temporal pressures. This is further illustrated by the accounts of other study participants, including nurses and integration managers, and discussed in greater detail in the following section.

As our data suggest, the separation of family and social environment should be also considered within the framework of care drain, given its multifaceted implications. On the one hand, there is the direct emotional burden associated with feelings of loneliness and sadness, particularly in the context of challenging and stressful situations such as high workloads, bullying, and illness. This issue is particularly exacerbated by the fact that, according to various reports, recruited professionals frequently experience a lack of support from colleagues and employers in difficult situations. On the other hand, the quote below shows a link between separation from the family environment and financial dependencies.

*Most colleagues, most people can't speak about it [racism], because they are in a bad situation. For example, they absolutely must send money to their families, or there are many people who also have sick relatives in their families, or something like this*. (Int 3, Interview, Pos. 8)^*^

Such dependencies frequently place individuals in vulnerable positions, as evidenced by our data. Among other factors, financial hardship and the fear of potential negative consequences—such as job loss—often hinder study participants from exercising their agency, as they remain silent about their circumstances.

*I have now been working for eight months as a nurse, and I earn the salary of a nursing assistant. With all the problems that I had, the administration refuses to pay my salary until my visa is cleared up. And in order to receive my visa, I needed an employment contract, which the clinic needed five months [to process]*. (Int 2, Diary, Pos. 15)

The above quote from Int 2 exemplifies how job dependency manifests within a particular political context. The interdependence between employment contracts and the issuance of visas exerts a significant influence on nurses involved in our study, as visa extensions are conditional upon the maintenance of a valid employment contract. The following section will explore this dynamic in relation to institutional dimensions.

*As soon as they make only the slightest sound, they are threatened [by the recruitment agency]. I have all of this in writing. They are threatened with having to go back to India again. […] Some of their papers have been taken away. Their partial recognition notices [Defizitbescheide] have been taken away, so that they cannot turn to another employer*. (Manag 3, Interview, Pos. 20)^*^

The aforementioned dependencies on employment and visa status, along with silencing dynamics, are in turn intensified by strategic and deliberate practices employed by recruitment agencies and employers. These practices often involve coercive tactics, including pressure and, in some cases, blackmail. This phenomenon is particularly evident in the accounts of the integration managers and in the document sample. Following the termination of employment contracts, affected study participants were formally requested—in accordance with additional clauses linked to the recognition procedure—to remit payments ranging from €2,000 to €15,000 to the employer within a matter of days. Additionally, the interviews revealed that nurses who participated in the study were subjected to considerable pressure to sign rental contracts immediately upon their arrival in Germany, despite their limited language proficiency and a lack of familiarity with the local legal system.

Manag 1's introductory statement, which is supported by accounts from other study participants, underscores that the recruitment of nurses is characterized by the narrative of a trade process that, within the context of neo-colonial power relations and on the basis of racialization, treats study participants as exploitable objects and degrades them to the status of commodities. Thus, the analytical framework employed in our study is grounded in global structural continuities marked by neo-colonial power relations, which encompass regimes of economic exploitation as well as racialized systems of knowing, being, and doing. Although the empirical material focuses on local, individual, and collective everyday experiences within the German context, a comprehensive interpretation and contextualization of these experiences requires situating them within the broader, historically embedded global conditions of inequality. Consequently, the effects and articulations of these global conditions are traceable—albeit frequently in indirect or nuanced forms—throughout the interviews, diary entries, and documents.

### 4.2 Institutional racism

*And so then they were given adhesion contracts, supplementary agreements that are not allowed—lawyers have also determined this to be the case—wherein it says: you are leaving us, or you have given notice to then and then. This means that the costs for recognition, according to the supplementary agreement of so and so, are now due. And then there are amounts such as 6,000, 12,000, 15,000. […] I know hardly anyone who does not have this adhesion contract. I now have some, three from [a German federal state], it's the same there too*. (Manag 3, Interview, Pos. 22)^*^

This quote by Manag 3, an independent integration manager who supports recruited nurses, refers to the routine granting of *Knebelverträge* (adhesion contracts)—agreements designed to bind one contracting party in a long-term and imbalanced contractual relationship, typically through the structuring of restrictive notice periods or termination conditions. In her view—as argued later in the same interview—these contracts lay the groundwork for (financial) dependency and facilitate exploitation. Another interviewee, who supports recruited nurses on a freelance basis, confirmed that this is a common practice. Accounts from other participants in the study illustrate that these institutional practices can come from both employing institutions (such as hospitals) and recruitment agencies. One participant provided us with documents (copies of contracts and correspondence with hospitals and authorities) after her interview to confirm her observations. Another study participant described cases in which recruited nurses were pressured to sign the above-mentioned contracts on arrival, even though, due to language barriers, the nurses did not understand them, or understood them insufficiently at the time of signing. There were also reports of hospitals “*taking away people's passports, so there's always the threat that if you don't do what we want, we'll send you back to your home country”* (Manag 2, Interview, Pos. 42)^*^. This quote illustrates the forms of degradation/dehumanization—such as restrictions on freedom of movement through the confiscation of participants' passports—that nurses face during the recruitment and hiring process, further increasing their dependency and vulnerability.

The accounts of the study participants illustrate how the degrading (institutional) practices described above, in the form of employment contracts, supplementary agreements, and the taking away of passports, are perpetuated in further regulations of the recognition process (*Anerkennungsphase*) for recruited nurses. One integration manager describes cases where recruited nurses, as part of the recognition process that they must go through to qualify and be paid the same as regular nurses, are initially paid as nursing assistants. However, she notes that they are often expected to carry out tasks that only regular nurses are entitled to do. In her view, this creates a gray area in which recruited nurses are assigned not only tasks beyond their formal area of competence, but also tasks that are particularly unpleasant (e.g., those that are physically demanding). Another study participant felt that recognition processes are deliberately prolonged in order to keep people in a lower pay grade for as long as possible. For example, it was reported that the human resources department of a hospital often did not issue the documents required for visa extensions on time, or that it issued them incorrectly, or even “lost” them, as some participants put it. This had led to delays in the recognition process and even work bans, resulting in months of lost income.

*The hospitals, and also, I'll just say, the nursing institutions, have consciously dragged this out so long so that they could continue to employ them as cheap labor. They have never even made the effort to enroll them in any kind of recognition course, whether it was the adaptation measure or professional recognition or language courses. The less they knew, the less language they had in order to defend themselves. So they were really dispatched and treated like old furniture, like old objects*. (Manag 3, Interview, Pos. 20)^*^

The accounts of Manag 3 summarize well the institutional practices that systematically perpetuate discrimination against recruited nurses. By denying them access to language courses and training, or by *taking advantage* of their lack of language and legal knowledge, the recruited nurses are made to perceive themselves as being dehumanized. They are treated as “objects” (commodified) and placed and kept in a dependent and exploitable position. As a result, the recruited nurses involved in the study are not seen and acknowledged as equal colleagues in day-to-day encounters and are also formally held below their actual qualifications. This is also reflected in the way they are treated by hospital management. For example, it was described in an interview that the recruited nurses are seen only as “*two more helping hands to do the work”* (Manag 2, Interview, Pos. 104)^*^, that when a nurse is seen as no longer fit, e.g., because of not having passed the recognition test, “*the clinic simply separates from [that nurse] and puts [them] on the street […] and then the next ones are taken”* (Manag 3, Interview, Pos. 20)^*^. This again illustrates the commodification approach, which is a common thread running through the accounts and diaries of other interviewees. The fact that this is done by managers indicates an institutional (re)production of degrading and ultimately dehumanizing tendencies toward recruited nursing staff.

Following on from this, a lack of “integration management” in hospitals was frequently reported both in the interviews and in the diaries. Study participants were impacted by the lack of a diversity-sensitive approach: there were few support services and there was often a lack of concepts for onboarding and supporting the recruited nursing staff. As a result, the interviewees often felt left alone by management, for example when it came to deciding which work-related tasks they should (already) take on as part of the recognition process. In this context, the lack of regulation was also criticized—in particular by the integration managers who participated in our study. Similar dynamics are described in more detail in the following section, which focuses on interpersonal encounters in nursing settings.

According to the nurses involved in the study, the (institutionalized) unequal treatment and dependencies described above, which occur routinely, can lead to further dynamics, within the hospitals, that put them at a systematic disadvantage. For example, Manag 3 explains that short-term changes were made in the working schedules of the recruited nursing assistants, and that this was done because of the assumption that they could be deployed quickly in the event of bottlenecks, or that that their off days or overtime could be canceled. In these instances, their lack of confidence in the German language was exploited, e.g., management would claim that there was simply a misunderstanding on the part of the employee, that the person was not actually off on the day in question. There were also reports of cases in which pregnant nurses were not protected in accordance with the relevant labor law requirements, as the quote shows:

*I have an example. There was a participant who had received an employment ban from her gynecologist on the ground of pregnancy, which is completely normal in nursing. Yes, it's also dangerous, and she went to her supervisor with it and the supervisor said that she had to keep working. Thus, the supervisor would not accept the employment ban*. (Manag 2, Interview, Pos. 42)^*^

Another participant described a similar situation: even though it was known that she was pregnant, she was not granted the days off that are prescribed for health and safety reasons. Instead, the management assigned her what she described as physically demanding tasks. In this way, the ban on employment in advanced pregnancy was disregarded.

The above accounts illustrate the simultaneity and intertwining of subtle processes of racialization, dehumanization, and commodification. These experiences were perceived by study participants as divisive and discriminatory, in the sense that they covertly create a zone where labor law standards that apply to white German care workers are overridden. Study participants are left with feelings of worthlessness, of being less worthy of protection.

### 4.3 Interpersonal racism

*The discrimination here is actually not in a big picture, but it's done in small, little ways. They [her colleagues] don't notice it, but they do it. And then when I leave the station, they will talk about me and then they will say: “She's ‘doof' [dumb]. She doesn't know how to speak the language. Why are they here?”* (Int 13, Interview, Pos. 24)

The accounts of Int 13, a female study participant, illustrate her experiences of racism with colleagues. She highlights how racial discrimination became evident through “*small, little ways”—*covertly, through gossip behind her back. Int 13 describes being perceived as someone who is less knowledgeable and is automatically presumed to be incapable of understanding or speaking the German language.

Similarly, other study participants described how experiences with racism include prevalent assumptions of incompetence. Int 1, a nurse, recounted a case that took place when she was in the recognition phase. Having practiced writing her nursing report, she asked a German colleague to check if it was correct. The colleague's response: “*You can't write properly anyway, let me do it [laughs]”* (Int 1, Interview, Pos. 16)^*^. This response illustrates how Int 1′s request was disregarded, not heard, ignored. A message of othering—of not seeing her as a knowledgeable and agentive subject—was implicitly conveyed. The accounts of another (experienced) nurse highlight how her professional knowledge—which she shared with the German trainee (Azubi) as part of her job—was actively devalued and disregarded, only to be accepted after the confirmation of another German colleague, who joined the team at a later date.

*[The trainee said:] “No, no, no, you don't know what you're talking about.” […] A [German] colleague said to me—she explicitly said, “after one day, after twenty-four hours, you can do that.” And she didn't want to listen to me. And so, I just didn't want to discuss it. I know, yes […] I first waited until our ward manager […] came. Then I asked in front of this student, who was a girl: “Yes, can you tell me again? Maybe I misunderstood earlier or at the time when I was being trained. Beginning at which hour or day could one do this […] check?” Then she said, “so after thirty-six hours or forty-eight hours.” Then the student was like: “Oh, so yes, then I misunderstood.”* (Int 16, Interview, Pos. 120)^*^

Some study participants emphasized the importance of how they are spoken to in encounters with colleagues. “The tone makes the music”^*^ were the words that Int 18 used when referring to an emergency case where he was addressed in a very unfriendly way. Similarly, Int 1 wrote in her diary: “*The colleague shouted and enforced authority over me […] ‘Where do you come from?”'* (Int 1, Diary, Pos. 13)^*^. Int 1 points to the covertly conveyed difference and subordination through tonality and speech, to the “innocuous” question that transmitted a message of non-belonging. Other study participants also described being shouted at and spoken to in a rude or condescending manner, as well as being confronted with “*böse*” (evil) looks by German colleagues, which was said to be intimidating. These experiences were perceived as an enforcement of division, as covertly solidifying a hierarchy of those who belong—who are knowledgeable and agentive—and those who do not belong—who are without knowledge and are encouraged to know their position and to limit their agency and speech.

Some study participants indicate that racism can also include more overt comments and actions from patients and, more significantly, from colleagues. Int 12 provided a detailed description of an incident in which a Filipino nurse was the subject of public bullying at the hands of her German co-worker. The latter was persistently seeking to identify mistakes and devalue the professional expertise of Int 12's colleague. In doing so, the German co-worker instigated verbal and tonal violence:

*That person [the German co-worker] kept on asking medical questions in German, and my Filipino colleague answered him correctly. But still, he was not satisfied with having tested her. He kept on finding some little mistakes with her and made it public to other workmates. He humiliated her because he said these things publicly and loudly. She was so embarrassed and mad because she knew that she was doing her best and she never even harmed any patients*. (Int 12, Diary, Pos. 5)

In the few cases where study participants dared to exercise their agency and name racist experiences, the *Schuld* (blame) was reversed by instrumentalizing language skills against them. For example, Int 3 described a situation in which she confronted a colleague's discriminatory behavior by taking the case to the *Betriebsrat* (works council). The colleague denied the accusation: “*No, I did not say that. [In a louder voice:] Int 3 cannot understand German”* (Int 3, Interview, Pos. 46)^*^. Rather than impartially considering the perspectives of both parties, the *Betriebsrat* representative undermined Int 3's credibility by questioning her proficiency in the German language and placed greater trust in the German co-worker's account, citing the co-worker's perceived politeness as justification:

“*Ah, are you sure you heard that?” I say: “Yes, I heard that.” “Ah, I think you didn't know […] I don't believe this, since she's so nice.” And I [laughs]—in this moment I was very nervous. Why would I lie about this? This is unbelievable*. (Int 3, Interview, Pos. 56)^*^

The lack of support from the *Betriebsrat* is also evident from colleagues, as described by Int 3 in the same interview. “*They [the colleagues] have always left me alone… I was like a ghost, you know? [slightly crying]”* (Int 3, Interview, Pos. 70)^*^. These were the words she used to capture the status of not being seen, of being left alone, ignored. Similarly, Int 2 recounted a case that she saw as demonstrating a lack of responsiveness from her boss. She reported facing difficulties to obtain a visa, which created uncertainty regarding her ability to return to work and maintain her income. She asked her boss to tell the responsible clinic administrator to contact the Job Center for clarification. However, her request was disregarded:

*I have heard back nothing [no response to her email]. He has ignored me, and I have still heard nothing from him, and there's no prediction as to when I'll return to work. I feel extremely frustrated, insignificant, and neglected by my employer […]and I've always worked*. (Int 2, Diary, Pos. 14)^*^

Accounts such as this one illustrate how interpersonal racism is intensified by the inaction and silence of white German colleagues. Silence and inaction mean that nurses affected by racial injustice must invest time and energy—resources that might otherwise be dedicated to patient care—into managing racist microaggressions, as described by Int 11:

*I feel like they are not trusting me or they are doubting me, even though I'm doing my best […] Everything that one does is wrong […] When I see this certain doctor or nurse who is toxic […] I cannot focus properly […] because […] what if I do something wrong? There are a lot of “what ifs” on my mind, so that I became too worried, and then sometimes my focus is disturbed*. (Int 11, Interview, Pos. 43)

This quote highlights not only the potential detrimental effects on the quality of patient care, but also the adverse impact on the psychological and emotional well-being of study participants subjected to racial injustice. The described experiences cumulatively add to an exhausting burden, as highlighted by Int 10 in her diary: “*I pray that I could handle this emotional damage”* (Pos. 60). Another study participant, who identified a recurring pattern in these experiences, describes the detrimental health effects associated with being treated differently, being ignored, or being socially isolated:

*I experience those things. Not only me, but also my colleagues. Yeah. Which is very traumatic, because as “Ausländer” [foreigners], we are alone here. And we feel that, you know, sometimes we feel that we do not belong to this place. Sometimes I just felt to myself, I told myself, will I work here a long time, or can I handle this situation? […] Mental health, it's really affected by those experiences*. (Int 10, Interview, Pos. 27)

In response to the recurring experiencing of being treated differently or of not being heard, nurses involved in the study reported how they began to refrain from articulating their concerns about experiencing racism. Some remained silent because they assumed that their counterparts would not have an appropriate understanding of their concerns. This was highlighted by Int 3:

*No, because I am alone, and when I clearly say something [in the sense of complaining] […] they say, that is not the truth. That's why I have not complained*. (Int 3, Interview, Pos. 46)^*^

Other participants remained silent, fearing negative consequences such as losing their jobs and/or visas, which would jeopardize their ability to provide financial support for their families, as articulated by participants Int 14 and Int 3:

*I was afraid that if I said something, then I'd be fired, or my words wouldn't count. I have no power*. (Int 14, Interview, Pos. 110)^*^

*Most colleagues […] don't speak about it [racism], because they are in a bad situation. For example, they absolutely must send money to their families, or there are many people who also have sick relatives in their families, or something like this. And then they're already under pressure*. (Int 3, Interview, Pos. 8)^*^

These accounts recount the structural dependencies that exist across situations—as outlined in the first section of the analysis—and shape interactions in nursing settings. The dependencies and vulnerabilities that emerge from these situations make it particularly difficult for study participants to defend themselves against racist discrimination. Instead, the nurses involved in our study countered their experiences with racism through extra work: “*We aren't talking about it […] We are just working […] Working and working and working”* (Int 10, Interview, Pos. 43). This was done in some cases to bolster their credibility, and in other cases in the hope of receiving less-degrading treatment.

## 5 Discussion

This article has explored the accounts of 18 internationally recruited nurses and three integration managers in Germany, who self-identify as “affected by racism,” regarding their professional experiences of racism in healthcare-related encounters.

The experiences shared by the study participants illustrate how racism manifests at interpersonal, institutional, and structural levels, which are intricately connected (Nazroo et al., 2020). As evident in the data, everyday interpersonal interactions embody structural racism in the form of microagressions (Sue, 2010). Microaggressions in our data express themselves non-verbally (dismissive looks) as well as through speech (questions and comments conveying difference and non-belonging) and tonality (speaking loudly), and they constitute “a form of systemic, everyday racism used to keep those at the racial margins in their place” (Pérez Huber and Solorzano, 2015, p. 298). These replicate the experiences of migrant nurses in England, as documented by Estacio and Saidy-Khan (2014). Study results further suggest that persistent racist knowledge can influence fellow staff members' ability to recognize and treat study participants as knowledgeable subjects—reflecting the colonial ideologies mentioned in the theoretical section regarding whose knowledge is considered valuable, and who is heard (Mills, 2007; Dotson, 2011). This is routinely expressed through assumptions about professional incompetence, as has already been documented in other studies (Likupe and Archibong, 2013; Beagan et al., 2023), through bullying (Wesołowska et al., 2020; Stevens et al., 2012) and through placement in inequitable and less desired work roles that involve heavier workloads, thus replicating the experiences of racism documented by Beagan et al. (2023).

The described dynamics of degradation and dehumanization are exacerbated by institutional practices, such as the issuance of employment contracts that create dependency, the prolongation of (“deskilling”) recognition processes to justify lower wages, the denial of opportunities for further training and education, disregard for minimum labor standards, and, in some cases, the confiscation of nurses' passports. All of this lays the groundwork for their commodification and exploitation. Lacking support from colleagues and managers, and reiterating concerns raised by others (Beagan et al., 2023), nurses in our study describe being left alone and not being heard in these precarious situations. Silence and inaction in response to racism function as a form of complicity, as they help maintain structural racism and implicitly convey additional messages of worthlessness and non-belonging to racialized nurses (Bouabdillah et al., 2021; Beagan and Etowa, 2009; Iheduru-Anderson et al., 2021).

The dependence on work visas—which, as Sharma (2020) argues, reproduce state racism by functioning as a technology of border regimes and by reinforcing racialized distinctions between “citizens” and “migrants”—as well as the separation from family and friends and financial obligations toward relatives abroad, creates further vulnerabilities. These conditions make it particularly difficult for study participants to defend themselves against racist discrimination (see also Ahlberg et al., 2022). Instead, experiences with racism were countered through extra work: this was done in some cases to bolster their credibility, and in other cases in the hope of receiving less-degrading treatment (see also Beagan et al., 2023; Ahlberg et al., 2022). This indicates that, as part of a broader structural racism (Mills, 2007; Nazroo et al., 2020), even coping and resistance strategies themselves are complicated by different modes of silencing/coerced self-silencing (Dotson, 2011).

Furthermore, our data suggest that processes of racialization can intensify commodification of (recruited) labor power and create a condition referred to as “precarity within precarity” (Carstensen et al., 2024) or the “racialization of precarity” (Ashiagbor, 2021). In such a situation, internationally recruited nurses not only endure exploitative labor conditions, but do so within segmented, racialized, work-related structures characterized by limited access to the full spectrum of human and citizenship rights, including labor rights. In other words, they arrive not in an empty or neutral space, but in spaces that are already “polluted” by racial power relations, and they are transitioned from a “zone of non-being” defined by “global coloniality” (non-Western periphery/Global South) to a “zone of non-being” marked by “internal colonialism” (within global metropolises of the West) (Grosfoguel et al., 2015, p. 638).

Experiences of racism, dehumanization, and commodification can result—as evidenced by the data—in elevated stress levels, pervasive feelings of anxiety, emotional exhaustion, and deterioration of (mental) health. These findings are consistent with studies by Hagey et al. (2001), Likupe and Archibong (2013), and Cottingham et al. (2018).

A closer examination of German history in the context of “labor” reveals striking parallels that suggest historically continuous interrelations between labor migration, political agreements, and, not least, systematic exploitation. While sociohistorical ruptures as well as migrant resistance and labor struggles (Pries and Dasek, 2017; Braeg, 2012) preclude a fully linear depiction of such a continuum, it can nevertheless be argued that—aside from the historically institutionalized reliance on foreign labor in the German Reich during the periods before, between, and during the World Wars—the guest worker agreements of the Federal Republic of Germany and the recruitment of contract workers in the GDR not only continue to have lasting effects (Ha et al., 2021), but also constitute a foundational pillar of German prosperity. These agreements form part of an ongoing pattern embedded within global, neo-colonial power relations. A notable historical example is the international recruitment of Korean nurses, in particular, between the 1950s and 1970s (Cho-Ruwwe, 2016). This case reveals clear parallels to the present day, especially with regard to the (non-)recognition of qualifications and experiences of direct discrimination (Cho-Ruwwe, 2016, 13ff.).

Taken together, the study participants' experiences are situated within a political and historical context shaped by racist colonial ideologies, discourses, and power structures that have persisted for centuries, operating both globally and nationally. From the perspective of Robinson's (1983) theory of racial capitalism, racism is a constitutive element of capitalist development, functioning as a fundamental organizing principle of the international division of labor and broader power hierarchies. In line with this framework, our data reveal how study participants—seeking to escape poverty, unemployment, and structural inequalities in their countries of origin—get entangled in new forms of structural inequality—through processes of racialization and commodification—in Germany. In other words, the experiences of internationally recruited nurses in Germany serve as a prism through which both the effects of global structures driving care drain and the resulting individual hardships and stress become visible.

### 5.1 Conclusion

Our study contributes to an empirically grounded understanding of experiences of racism that have been reported by internationally recruited nurses and integration managers in Germany. It thus allows the reader to consider how racism and racialization intersect the processes of dehumanization and commodification, and it thereby offers a more nuanced perspective on racialized labor relations in transnational care work and on the dynamics of care drain (Kaelin, 2011). What distinguishes this approach, both within the German context and beyond, is its focus on the intersection of racialization—as a process that structures global (labor) hierarchies of worthiness (Fanon, 2008; Robinson, 1983)—and commodification, understood here as the capitalist reduction of laboring bodies to units of exchange (Ashiagbor, 2021). Instead of approaching racialization and commodification as separate or sequential phenomena, our analysis highlights their mutually reinforcing intersections: commodification is frequently shaped by racializing logics/dynamics, just as racialization is often mobilized to serve commodifying purposes. As pointed out by Ruth Wilson Gilmore in an interview with Mizue Aizeki (Aizeki, 2023), capitalism requires inequality, and racism/racialization helps to enshrine that inequality (see also Robinson, 1983).

Although our findings are context-specific, the conceptual framework developed in this paper is transferable to other labor sectors where racialization and commodification may intersect, such as domestic work, agriculture, and logistics. Existing literature from these sectors supports the broader applicability of the study's core arguments (e.g., Carstensen et al., 2024; Fraser, 2016). Moreover, our analysis echoes critiques of the extractive nature of global (nursing) care chains and the devaluation and exploitation of care work under global capitalism (Yeates, 2009). As such, it can serve as a basis for fostering constructive dialogue with healthcare providers and policymakers. It underscores the urgent need for systemic transformation within the healthcare sector, particularly through the adoption of anti-racist policies. Such policies must confront racism at interpersonal, institutional, and structural levels, recognizing how these dimensions intersect and reinforce one another. Key areas for intervention include improving working conditions in nursing, implementing robust monitoring and accountability mechanisms for recruitment agencies and healthcare institutions, moving from cultural competency education to anti-racist education in nursing curricula, optimizing onboarding and mentoring programs that address the specific needs of internationally recruited nurses, and advancing transdisciplinary and transnational research initiatives.

The application of a qualitative study design—particularly the combination of interviews and diaries—enabled a more comprehensive exploration of racialization processes and subtle, otherwise hard-to-detect dynamics such as dehumanization and commodification, along with their intersectional manifestations. The diaries, in particular, provided a means to capture participants' moments of self-reflexivity and offered rich, contextualized data about routines and lived experiences (Theodosius, 2008), which might be overlooked or remain inaccessible through interviews alone.

Finally, it is important to emphasize that internationally recruited nurses are active agents with their own capacity for action. They develop strategies to confront racial injustice and to build resistance and solidarity. This is evident in both the interviews and the diary entries, as well as in the international literature (Ramamurthy et al., 2022). Participants' agency and resistance will be examined in greater depth in a follow-up publication, as the present manuscript primarily focuses on the complex intersections of racialization and commodification.

### 5.2 Limitations

Our study is limited by having a relatively small and heterogeneous sample, which includes individuals subjected to various forms of racism—such as anti-Black racism, anti-Asian racism, and racism against Muslims. However, this heterogeneity also constitutes a methodological strength, as it enables an examination of racism across multiple racialized groups and facilitates an exploration of the broader processes of racialization. Another limitation arises out of the study‘s primary focus on racism. This emphasis is intended not to diminish the significance of other forms of discrimination, but rather to underscore the necessity of dedicating focused analytical attention to racism. It is important to acknowledge, however, that participants' experiences were shaped not solely by racism, but also by their intersecting social locations—including, but not limited to, gender, class, religion, and migration status. This is a fertile field for further exploration. Finally, it is important to note that this paper does not include the perspectives of racialized nurses who are socialized and professionally trained in Germany, as the composition of the sample did not permit their representation.

## Data Availability

The datasets presented in this article are not readily available because of legal, ethical, and privacy restrictions. Requests to access the datasets should be directed to gangarova@dezim-institut.de.

## Footnotes

^1^The German expression “Ich hätte gerne fünf Stück” is difficult to translate precisely into English. Our rendering—“I'd like five of them”—represents a stylistic compromise. It does not fully capture the objectifying undertone of the original, in which the referents (e.g., nurses) are implicitly treated as countable items/pieces (“Stück”).

^2^For decolonial theorists like Grosfoguel, the Global South is not merely a geographical location but a positionality within the global order. It refers to populations, territories, and knowledge systems that have been historically marginalized, colonized, or exploited—even when located in the Global North, such as marginalized communities within Europe.
